# Regulation of Regenerative Periodontal Healing by NAMPT

**DOI:** 10.1155/2013/202530

**Published:** 2013-10-28

**Authors:** Marjan Nokhbehsaim, Sema Keser, Andreas Jäger, Søren Jepsen, James Deschner

**Affiliations:** ^1^Experimental Dento-Maxillo-Facial Medicine, Center of Dento-Maxillo-Facial Medicine, University of Bonn, 53111 Bonn, Germany; ^2^Clinical Research Unit 208, Center of Dento-Maxillo-Facial Medicine, University of Bonn, Welschnonnenstraße 17, 53111 Bonn, Germany; ^3^Department of Orthodontics, University of Bonn, 53111 Bonn, Germany; ^4^Department of Periodontology, Operative and Preventive Dentistry, University of Bonn, 53111 Bonn, Germany

## Abstract

Periodontitis is an inflammatory disease characterized by destruction of the tooth-supporting tissues. Obese individuals have an increased risk of periodontitis, and elevated circulating levels of nicotinamide phosphoribosyltransferase (NAMPT) may be a pathomechanistic link between both diseases. Recently, increased levels of NAMPT have also been found in patients with periodontitis, irrespective of the presence of obesity. This in vitro study sought to examine the effects of NAMPT on the regenerative capacity of human periodontal ligament (PDL) cells and, thereby, periodontal healing. PDL cells treated with enamel matrix derivative (EMD), which was used to mimic regenerative healing conditions in vitro, were grown in the presence and absence of NAMPT for up to 14 d. EMD stimulated significantly (*P* < 0.05) the expression of growth factors and their receptors, matrix molecules, osteogenesis-associated factors, and wound closure and calcium accumulation. In the presence of NAMPT, all these stimulatory effects were significantly (*P* < 0.05) reduced. In conclusion, the beneficial effects of EMD on a number of PDL cell functions critical for periodontal regeneration are counteracted by NAMPT. Enhanced levels of NAMPT, as found in obesity and periodontal inflammation, may compromise the regenerative capacity of PDL cells and, thereby, periodontal healing in the presence of EMD.

## 1. Introduction

Periodontitis is a chronic multifactorial inflammatory disease that is characterized by the progressive destruction of the tooth-supporting tissues, such as the periodontal ligament (PDL). Pathogenic microorganisms embedded in a biofilm on the tooth surface can trigger local synthesis of proinflammatory mediators and matrix-degrading proteases by infiltrating and resident cells of the periodontium. As a consequence of the exaggerated immunoinflammatory and proteolytic processes, the periodontal tissues are subjected to degradation and resorption, which can result in periodontal pocket formation and even tooth loss [1–4]. The National Health and Nutrition Examination Survey III that analyzed the health and nutritional status in the United States revealed that approximately half of the US population aged ≥30 years suffers from periodontitis [5]. It is widely accepted that periodontitis has a significant negative impact on physical, psychological, and social aspects of life and also affects systemic health in affected individuals [6–8]. 

The main goal of periodontal therapy is to arrest the inflammatory and tissue-destructive processes by reducing or eliminating the pathogenic microorganisms in the periodontal pockets [9]. Conventional periodontal treatment comprises nonsurgical or surgical debridement, sometimes applied in combination with antibiotics, and achieves periodontal healing mainly by repair [10]. Regenerative healing can be stimulated by the application of bioactive molecules, such as enamel matrix derivative (EMD), during periodontal surgery [11–13]. A great number of in vitro studies have demonstrated that EMD stimulates the synthesis of growth factors, such as vascular endothelial growth factor (VEGF) and transforming growth factor (TGF) *β*1, matrix molecules, such as collagen type I (COL1) and periostin (POSTN), osteogenesis-related factors, such as runt-related transcription factor (RUNX) 2, calcium deposition, and in vitro wound healing [14, 15]. It is widely accepted that the regeneration-promoting activities of EMD are induced at least in part by these mechanisms. Moreover, it has been shown that the beneficial effects of EMD on periodontal regeneration are mediated, at least partially, by bone morphogenetic protein (BMP) and TGF*β*, because fractions of EMD contain BMP- and TGF*β*-like activity and EMD stimulates BMP and TGF*β* synthesis in periodontal cells [16–19]. Binding of TGF*β* and BMP to their receptor complexes triggers SMAD (sma- and mad-related protein) and non-SMAD signaling cascades [20, 21]. 

Regeneration of periodontal tissues is often an unpredictable challenge due to a number of as yet unidentified local and systemic factors that can modulate healing processes. Previous studies suggest that microbial, inflammatory, and biomechanical signals can interfere with the beneficial effects of EMD on periodontal cells, emphasizing the critical role of the cell environment for optimal periodontal regeneration [22–27]. In addition, recent meta-analyses have revealed that obesity, metabolic syndrome, and diabetes mellitus are significantly associated with periodontitis, and it has been suggested that adipokines might be a critical pathomechanistic link in these associations [28–31]. Adipokines are cytokines released from the adipose tissue and regulate food intake and energy expenditure but also immunoinflammatory processes [32, 33]. Since the serum levels of a number of proinflammatory adipokines, such as nicotinamide phosphoribosyltransferase (NAMPT), are increased in obesity and some obesity-related diseases, it has been speculated that such adipokines could enhance periodontal inflammation and, thereby, increase the risk of periodontitis or compromise periodontal healing in obese individuals [34–38].

NAMPT is mainly produced by macrophages and adipocytes in the adipose tissue, triggers NF*κ*B activation, and elicits synthesis of inflammatory mediators [39]. Increased serum levels of NAMPT have been found in obesity, metabolic syndrome, type 2 diabetes, atherosclerosis, and other diseases [34–36]. Therefore, elevated NAMPT levels could be one mechanism, whereby these diseases contribute to the initiation and progression of periodontitis. Interestingly, NAMPT is also present at high levels in gingival crevicular fluid (GCF), gingival tissues, and serum from patients afflicted with periodontitis, irrespective of the presence of obesity [40–42]. These observations indicate that NAMPT is also produced locally in the periodontium and may play a role in the etiopathogenesis of periodontitis. Our previous experiments have demonstrated that periodontal pathogens upregulate NAMPT in periodontal cells and, additionally, that NAMPT could contribute to periodontal inflammation and destruction by stimulating periodontal cells to produce proinflammatory and proteolytic molecules [43, 44]. However, whether NAMPT also interferes with periodontal healing is as yet unknown. Therefore, this in vitro study sought to examine whether the regenerative capacity of periodontal cells in the presence of EMD is modulated by NAMPT. A better understanding of the interactions between regenerative molecules and local as well as systemic factors may help better predict and even improve the outcome of currently applied periodontal treatment approaches. 

## 2. Materials and Methods

### 2.1. Culture and Treatment of Cells

**Figure 1:**
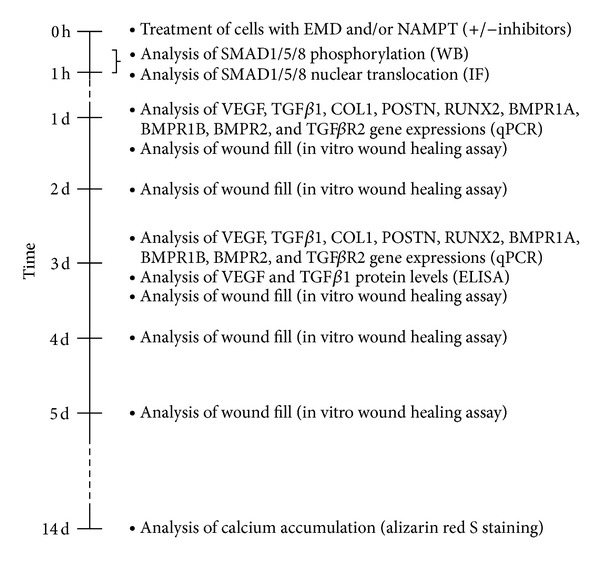
Study design flowchart. EMD: enamel matrix derivative; NAMPT: nicotinamide phosphoribosyltransferase; SMAD: sma- and mad-related protein; WB: western blot; IF: immunofluorescence; VEGF: vascular endothelial growth factor; TGF*β*1: transforming growth factor *β*1; COL1: collagen type I; POSTN: periostin; RUNX2: runt-related transcription factor 2; BMPR1A, 1B, or 2: bone morphogenetic protein receptor 1A, 1B, or 2; TGF*β*R2: TGF*β* receptor 2; qPCR: quantitative polymerase chain reaction; ELISA: enzyme-linked immunosorbent assay.

PDL cells from 18 periodontally healthy donors, who underwent tooth extraction for orthodontic reasons, were used for the experiments. Informed consent and approval of the Ethics Committee of the University of Bonn were obtained. The cells were grown in Dulbecco's minimal essential medium (DMEM, Invitrogen, Karlsruhe, Germany) supplemented with 10% fetal bovine serum (FBS, Invitrogen), 100 units penicillin, and 100 *μ*g/mL streptomycin (Invitrogen) at 37°C in a humidified atmosphere of 5% CO_2_. Cells between 3rd and 5th passage were seeded (50,000 cells/well) on culture plates and grown to 80% confluence. One day prior to the experiments, the FBS concentration was reduced to 1%. Medium was changed every second day. In order to simulate regenerative conditions in vitro, PDL cells were treated with EMD (Emdogain, Straumann, Freiburg, Germany) in the presence and absence of NAMPT. As in our previous studies, EMD was applied at a concentration of 100 *μ*g/mL because this concentration had been used by several investigators before and ensured that our data were comparable with those of other studies [24–27]. In order to investigate the effects of NAMPT, various concentrations of this adipokine (30, 100, and 300 ng/mL; Biomol, Hamburg, Germany) were added to cells. In our experiments, the standard concentration of NAMPT was 100 ng/mL. This concentration correlates well with the studies by Pradeep and coworkers, who have reported a NAMPT concentration of 98.32 ng/mL in gingival crevicular fluid [40–42]. However, these studies only included a small number of patients, so that even higher concentrations can be expected in some individuals and were therefore also studied in a subset of our experiments. In the present study, cells were exposed to EMD and/or NAMPT for up to 14 d (Figure 1). In order to analyze the intracellular mechanisms used by NAMPT to modulate the actions of EMD, cells were preincubated with specific inhibitors against NF*κ*B (pyrrolidine dithiocarbamate, PDTC; 10 *μ*M; Calbiochem, San Diego, CA, USA), MEK1/2 (U0126; 10 *μ*M; Calbiochem), JNK (SP600125; 10 *μ*M; Calbiochem), and p38 (SB203580; 10 *μ*M; Calbiochem) signaling pathways 1 h before experiments.

### 2.2. Real-Time PCR

RNA was extracted by using an RNA extraction kit (Qiagen, Hilden, Germany), and a total of 1 *μ*g of RNA was reverse transcribed using iScriptTM Select cDNA Synthesis Kit (Bio-Rad Laboratories, Munich, Germany) at 42°C for 90 min followed by 85°C for 5 min. Expression of VEGF, TGF*β*1, and its receptor TGF*β*R2, BMP receptors (BMPR1A, BMPR1B, and BMPR2), COL1, POSTN, and RUNX2 was detected by real-time PCR using the iCycler iQ detection system (Bio-Rad Laboratories), SYBR Green (Bio-Rad Laboratories), and specific primers (QuantiTect Primer Assay, Qiagen) (Figure 1). One *μ*L of cDNA was amplified as a template in a 25 *μ*L reaction mixture containing 12.5 *μ*L 2x QuantiFast SYBR Green PCR Master Mix (Qiagen), 2.5 *μ*L of primers, and 9 *μ*L deionized water. The mixture was heated initially at 95°C for 5 min and then followed by 40 cycles with denaturation at 95°C for 10 s and combined annealing/extension at 60°C for 30 s. Glyceraldehyde-3-phosphate dehydrogenase was used as an endogenous control. The data were analyzed by the comparative threshold cycle method. 

### 2.3. ELISA

The protein levels of VEGF and TGF*β*1 in the supernatants of PDL cells were analysed by commercially available enzyme-linked immunosorbent assay (ELISA) kits (R&D Systems, Minneapolis, MN, USA) according to the manufacturer's instructions (Figure 1). The absorbance was measured by using a microplate reader (PowerWave x, BioTek Instruments, Winooski, VT, USA) at 450 nm. The data were normalized by cell number that was determined with an automatic cell counter (Moelab, Hilden, Germany).

### 2.4. Alizarin Red S Staining

The calcium accumulation in PDL cell cultures following 14 d of treatment with EMD and/or NAMPT was analyzed by using alizarin red S staining (Merck KGaA, Darmstadt, Germany) and cetylpyridinium chloride (Sigma-Aldrich Chemie, Munich, Germany), as described in our previous studies (Figure 1) [24, 25]. Briefly, cell monolayers were washed with PBS, fixed in 4% paraformaldehyde (Merck KGaA) for 10 min, and washed again with deionized water. Afterwards, cells were incubated with 40 mM alizarin red S (pH 4.2) for 15 min, rinsed with deionized water, and subsequently incubated with 10% (w/v) cetylpyridinium chloride in 10 mM sodium phosphate (pH 7.0) in order to extract the alizarin red S that was retained in the cell cultures. Following 15 min of incubation, cells were detached from the well surface. The detached cells and cetylpyridinium chloride were collected and centrifuged at 20,000 g for 10 min. Afterwards, the pellets were discarded and the supernatants that contained the extracted stain were transferred into a 96-well plate to analyze the absorbance at 562 nm using a microplate reader.

### 2.5. *In Vitro*  Wound Healing

In order to examine the effect of NAMPT on wound fill, an in vitro wound healing model was used, as in our previous experiments [24, 25]. Briefly, cells were grown until confluence and 3 mm wide wounds were created in a standardized manner in the cell monolayers. The wounded cell monolayers were treated with EMD and, simultaneously, with various concentrations of NAMPT (0, 30, 100, and 300 ng/mL) for 5 d. At every day, the wounds were documented by inverse microscopy (Axiovert 25 C, 5x objective, Carl Zeiss, Oberkochen, Germany) and digital photography (Kodak DC 290, Kodak, Stuttgart, Germany) (Figure 1). Measurement and analysis of the wound widths were performed with special software (Alpha DigiDoc 1000, Alpha Innotech, San Leandro, CA).

### 2.6. Immunofluorescence

PDL cells were fixed with 4% paraformaldehyde in PBS pH 7.4 for 10 min, washed with PBS, and treated with 0.1% Triton X-100 (Sigma-Aldrich, Munich, Germany) for 5 min. Then, cells were washed again and blocked with nonfat dry milk (Bio-Rad Laboratories) for 1 h. After washing, cells were incubated with a rabbit anti-SMAD1/5/8 antibody (Santa Cruz Biotechnology, Santa Cruz, CA, Germany) for 90 min and with CY3-conjugated goat anti-rabbit IgG (Abcam, Cambridge, MA, USA) for 45 min. Cells were observed under a 20x objective using an Axioplan 2 imaging microscope (Carl Zeiss). The images were captured with a PVCAM camera and the VisiView capturing software (Visitron Systems, Puchheim, Germany) (Figure 1).

### 2.7. Western Blot Analysis

Phosphorylation of SMAD1/5/8 was analyzed from whole lysate (20 to 40 *μ*g protein) of cells treated with EMD in the presence or absence of NAMPT (Figure 1). Cells were washed twice with ice cold PBS and collected in RIPA buffer (Sigma-Aldrich) supplemented with protease inhibitors (Sigma-Aldrich). Equal amounts of protein were resolved through SDS-polyacrylamide gel electrophoresis and transferred to nitrocellulose membranes (Bio-Rad Laboratories). The membranes blocked with 5% nonfat milk were probed with a specific rabbit anti-pSMAD1/5/8 antibody (Cell Signalling Technology, Danvers, MA, USA). The binding of the primary antibody was revealed with horseradish peroxidase- (HRP-) labeled goat anti-rabbit IgG (Jackson ImmunoResearch, Suffolk, UK). Lightning chemiluminescence reagent (Thermo Fisher Scientific, Rockford, IL, USA) was used as an HRP substrate. All blots were reprobed with rabbit anti-SMAD1/5/8 (Santa Cruz Biotechnology) and mouse anti-*β*-actin antibodies (abcam, Cambridge, UK) to assure equal input of proteins. 

### 2.8. Statistical Analysis

All experiments were performed in triplicate and repeated at least twice. Mean values and standard errors of the mean (SEM) were calculated. Parametric (*t* test and ANOVA followed by the post hoc Tukey's test) and nonparametric tests (Wilcoxon and Mann-Whitney *U*-tests) were applied for statistical analysis by using the IBM SPSS Statistics 20 software. Differences between groups were considered significant at *P* < 0.05.

## 3. Results 

### 3.1. Regulation of EMD Effects on Growth Factors by NAMPT

**Figure 2:**
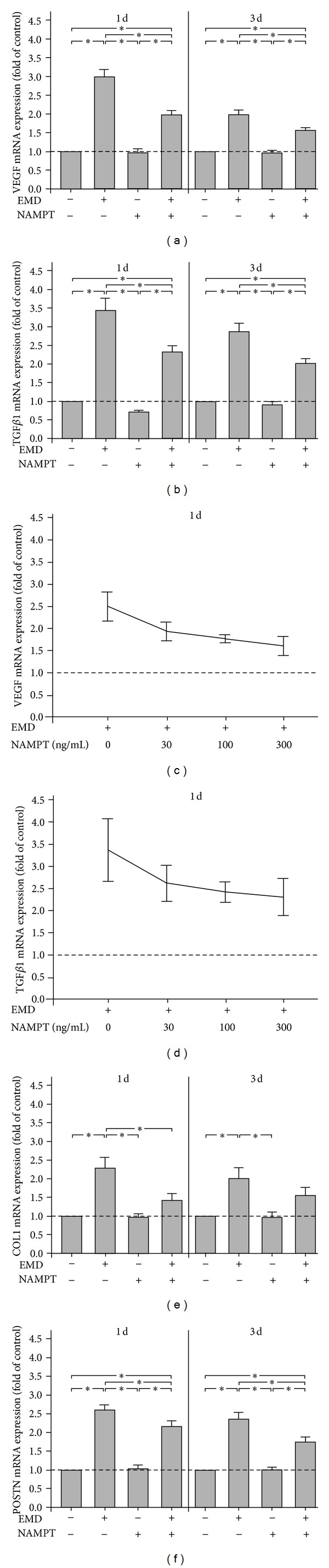
Effect of EMD on the VEGF (a), TGF*β*1 (b), COL1 (e), and POSTN (f) mRNA expressions in the presence and absence of NAMPT (100 ng/mL) at 1 d and 3 d. Untreated cells served as control. All experiments were performed in triplicate and repeated at least twice. Mean ± SEM (*n* = 18); *significant (*P* < 0.05) difference between groups. Effect of various concentrations (0, 30, 100, and 300 ng/mL) of NAMPT on the VEGF (c) and TGF*β*1 (d) mRNA expressions in EMD-treated cells at 1 d. All experiments were performed in triplicate and repeated at least twice. Mean ± SEM (*n* = 9).

Since the regeneration-promotive effects of EMD are at least partly mediated by upregulation of growth factors, we first sought to determine whether these EMD actions on PDL cells are modulated by NAMPT. As expected from previous studies, EMD caused a significant upregulation of VEGF and TGF*β*1 in PDL cells at 1 d and 3 d. However, the EMD-stimulated VEGF und TGF*β*1 expressions were significantly reduced in the presence of NAMPT at both time points, as shown in Figures 2(a) and 2(b). Interestingly, NAMPT did not induce a significant downregulation of the constitutive VEGF and TGF*β*1 expressions (Figures 2(a) and 2(b)). Dose response experiments revealed that the inhibitory effects of NAMPT on the EMD-stimulated VEGF and TGF*β*1 upregulation were similar over a wide range of concentrations (Figures 2(c) and 2(d)). By preincubation of cells with a specific inhibitor against the JNK signaling pathway, the inhibitory effects of NAMPT on the EMD-induced VEGF and TGF*β*1 expressions were completely blocked at 1 d. However, inhibition of NF*κ*B, MEK1/2, or p38 signaling did not interfere with the actions of NAMPT at this time point (data not shown).

The inhibitory effects of NAMPT on the EMD-stimulated VEGF and TGF*β*1 expressions were also found at protein level, as analyzed by ELISA after 3 d (Table 1).

### 3.2. Modulation of EMD Actions on Matrix Molecules by NAMPT

EMD also stimulates periodontal regeneration by upregulation of matrix molecules. Therefore, we next studied whether actions of EMD on matrix molecules are modulated by NAMPT. EMD increased significantly the expression of COL1 and POSTN at 1 d and 3 d, but these stimulatory effects were significantly inhibited by NAMPT for COL1 at 1 d and for POSTN at 1 d and 3 d (Figures 2(e) and 2(f)). NAMPT had no regulatory effects on the spontaneous expression of COL1 and POSTN (Figures 2(e) and 2(f)).

### 3.3. Effects of NAMPT on EMD-Stimulated Mineralization

**Figure 3:**
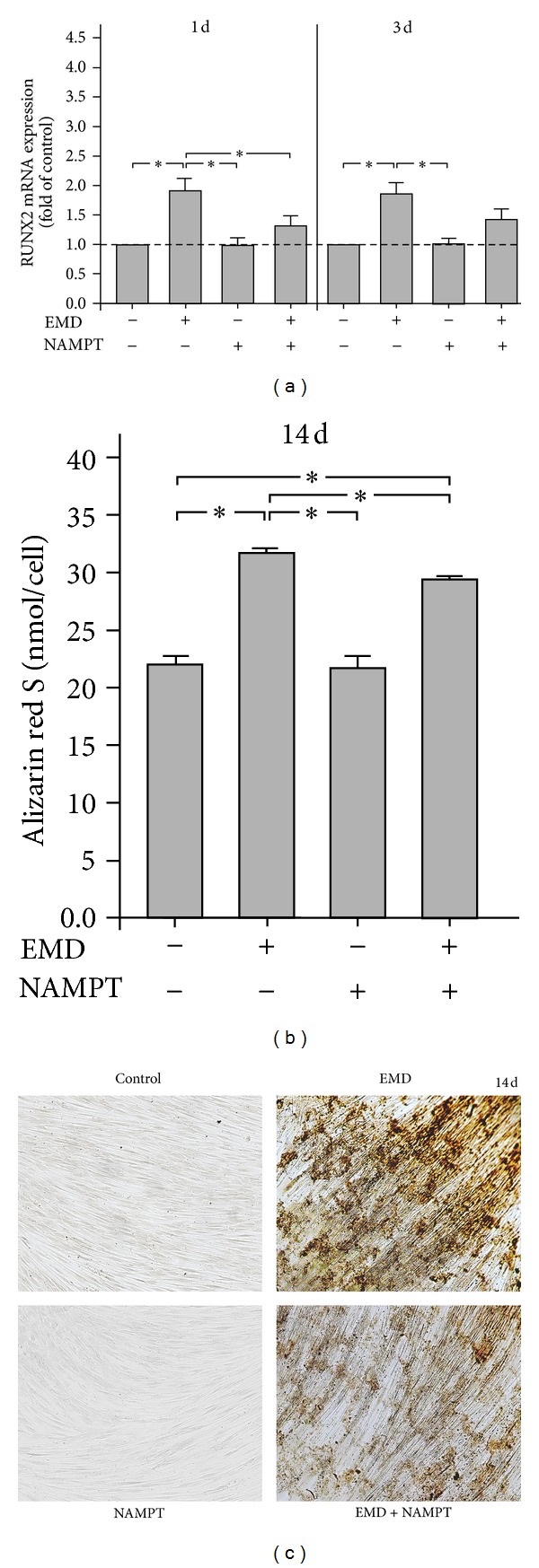
Effect of EMD on the RUNX2 (a) mRNA expression in the presence and absence of NAMPT (100 ng/mL) at 1 d and 3 d. Untreated cells served as control. All experiments were performed in triplicate and repeated at least twice. Mean ± SEM (*n* = 18); *significant (*P* < 0.05) difference between groups. Effect of EMD on the calcium accumulation in the presence and absence of NAMPT (100 ng/mL) in cultures of PDL cells ((b) and (c)). Untreated cells served as control. The calcium accumulation was analysed by alizarin red S staining, visualized by microscopy (c), and quantified by elution with cetylpyridinium chloride (b) at 14 d. All experiments were performed in triplicate and repeated at least twice. Mean ± SEM (*n* = 12); *significant (*P* < 0.05) difference between groups. Images from one representative donor are shown.

Next, we wondered whether NAMPT would also interfere with the stimulatory effects of EMD on osteogenesis. As expected, EMD stimulated significantly the expression of RUNX2, an osteogenesis-associated transcription factor, at 1 d and 3 d (Figure 3(a)). Interestingly, the EMD-induced RUNX2 expression was also significantly downregulated by NAMPT, which reached significance at 1 d (Figure 3(a)). Furthermore, treatment of cells with EMD for 14 d caused a pronounced calcium accumulation in cell cultures, as determined by alizarin red S staining (Figures 3(b) and 3(c)). When cells were simultaneously treated with EMD and NAMPT, the calcium accumulation was significantly reduced as compared to EMD-stimulated cultures in the absence of NAMPT (Figures 3(b) and 3(c)).

### 3.4. Effects of NAMPT on EMD-Induced Wound Healing

**Figure 4:**
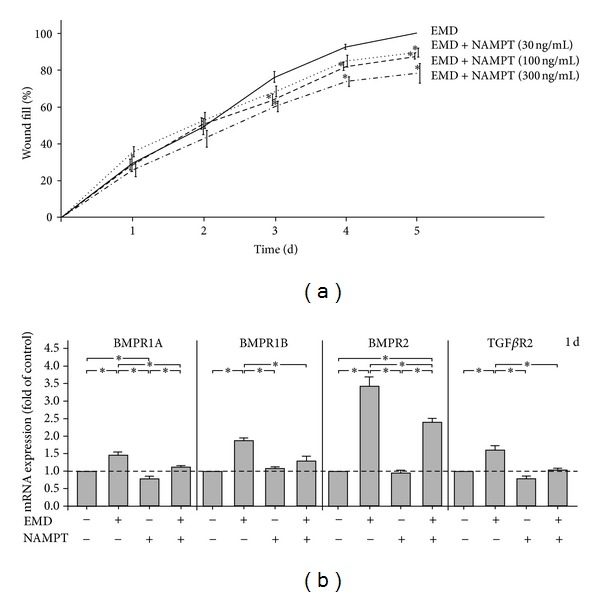
Effect of various concentrations (0, 30, 100, and 300 ng/mL) of NAMPT on the EMD-induced PDL cell wound fill rate (a). The wound closure, that is, the percentage of fill of the initially cell free zones created by wounding, were analysed over 5 d. All experiments were performed in triplicate and repeated at least twice. Mean ± SEM (*n* = 12); *significantly (*P* < 0.05) different from EMD-treated cells in the absence of NAMPT. Effect of EMD on the expression of BMPR1A, BMPR1B, BMPR2 and TGF*β*R2 in the presence and absence of NAMPT (100 ng/mL) at 1 d (b). Untreated cells served as control. All experiments were performed in triplicate and repeated at least twice. Mean ± SEM (*n* = 18); *significant (*P* < 0.05) difference between groups.

NAMPT also caused a dose-dependent inhibition of the EMD-stimulated wound healing (Figure 4(a)). The EMD-induced wound closure was significantly reduced by 100 ng/mL of NAMPT and even more pronounced by 300 ng/mL of NAMPT at 3, 4, and 5 d, whereas 30 ng/mL of NAMPT had only a significantly inhibitory effect at 5 d (Figure 4(a)). 

### 3.5. Effects of NAMPT on BMPRs and TGF*β*Rs in the Presence and Absence of EMD

At least some actions of EMD seem to be mediated by BMP or TGF*β* because EMD has been shown to possess BMP- and TGF*β*-like activity and, additionally, to upregulate these molecules in periodontal cells. We therefore also examined the effects of NAMPT on BMPRs and TGF*β*Rs. As shown in Figure 4(b), EMD increased significantly the constitutive expression of BMPR1A, BMPR1B, BMPR2, and TGF*β*R2 at 1 d. However, NAMPT caused a significant downregulation of the EMD-stimulated expression of these receptors at this time point (Figure 4(b)). Similar results were found for 3 d (data not shown). In the absence of EMD, a significant inhibition by NAMPT was only found for BMPR1A (Figure 4(b)). 

### 3.6. Inhibition of SMAD Signaling by NAMPT in EMD-Treated Cells

**Figure 5:**
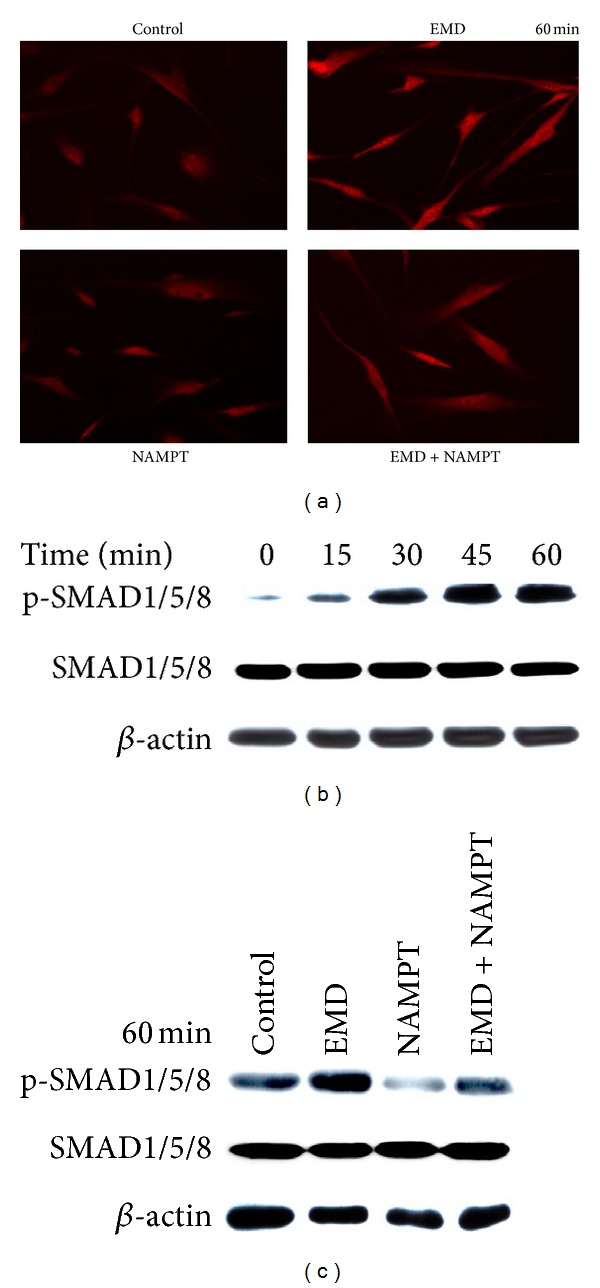
Effect of EMD on the nuclear translocation of SMAD1/5/8 in the presence and absence of NAMPT (100 ng/mL) at 60 min, as determined by immunofluorescence (a). Untreated cells served as control. Stimulation of SMAD1/5/8 phosphorylation by EMD over 60 min, as analyzed by immunoblotting (b). Effect of EMD on SMAD1/5/8 phosphorylation in the presence and absence of NAMPT (100 ng/mL) at 60 min, as examined by immunoblotting (c). All experiments were performed in triplicate and repeated at least twice. Images and blots from one representative donor are shown.

Next, we sought to unravel intracellular mechanisms, whereby NAMPT abrogates regeneration-promotive actions of EMD. As shown in Figure 5(a), EMD caused a pronounced accumulation of SMAD1/5/8 in the nucleus of EMD-treated cells at 60 min. However, in the presence of NAMPT, this EMD-stimulated nuclear translocation of SMAD1/5/8 was reduced. In the absence of EMD, no obvious effect of NAMPT on the SMAD1/5/8 nuclear translocation was observed (Figure 5(a)). Further experiments revealed that EMD stimulated the phosphorylation of SMAD1/5/8 in a time-dependent manner, as evidenced by immunoblotting (Figure 5(b)). As shown in Figure 5(c), the EMD-stimulated SMAD1/5/8 phosphorylation was inhibited in the presence of NAMPT.

## 4. Discussion

The present study demonstrates for the first time that regeneration-promotive actions of EMD on a great number of PDL cell functions critical for periodontal regeneration are jeopardized by NAMPT. The findings of this study suggest that increased levels of NAMPT, as found in obesity and periodontal inflammation, may compromise the regenerative capacity of PDL cells and, thereby, periodontal healing in the presence of EMD. 

 NAMPT abrogated the stimulatory effects of EMD on VEGF and TGF*β*1, COL1, in vitro wound healing, and osteogenic differentiation. VEGF is an essential growth factor, which supports wound healing by its regulatory effects on vascular permeability, influx of inflammatory cells into the site of injury, migration and proliferation of preexisting endothelial cells, and the recruitment of marrow-derived endothelial progenitor cells to the local wound site [45]. In addition, VEGF seems to play an important role in the modulation of bone remodeling by attracting endothelial cells and osteoclasts and by stimulating osteoblast differentiation [46]. TGF*β*, another important growth factor, comprises three isoforms and also promotes wound healing like VEGF. TGF*β*1 supports migration of wound keratinocytes and, thereby, successful reepithelialization [47]. Moreover, it induces fibroblasts to deposit new extracellular matrix proteins, which promotes cell and vascular in-growth [48]. In addition to growth factors, matrix molecules, such as COL1 and POSTN, are also critical for periodontal homeostasis and healing. Like COL1, POSTN is also strongly expressed in the human PDL and regulates cell-matrix interactions as well as cell adhesion, proliferation, and differentiation [49, 50]. Furthermore, periostin also aids to disperse mechanical forces applied to the PDL [50, 51]. Interestingly, NAMPT also counteracted the stimulatory effects of EMD on the in vitro wound healing. Following periodontal treatment, periodontal wounds need to be repopulated with cells to achieve periodontal healing [52]. When EMD-treated cells were exposed to NAMPT, the wound closure was dose-dependently delayed, indicating that NAMPT may also inhibit critical cell functions, such as proliferation and migration, under regenerative conditions. PDL cells can acquire an osteoblastic phenotype, and a number of studies have proven that EMD stimulates expression of osteogenesis-associated factors, such as RUNX2, and calcium deposition, an early marker of matrix mineralization, from PDL cells [14]. As expected, EMD upregulated RUNX2 and induced calcium deposition in the present study. In the presence of NAMPT, the stimulatory effects of EMD on these osteogenesis-related processes were reduced. Although NAMPT inhibited significantly the EMD-induced RUNX2 and collagen expressions at 1 day, the inhibitory effects of NAMPT did not reach significance at 3 days due to the pronounced standard deviations. However, including a higher number of donors in these experiments might have resulted in statistically significant changes also at 3 days. Nevertheless, our findings suggest that NAMPT interferes with both the EMD-induced effects on both periodontal soft and hard tissue regeneration.

We also sought to unravel the mechanisms, whereby NAMPT modulates the actions of EMD. Fractions of EMD contain BMP- and TGF*β*-like activity and EMD also upregulates BMP and TGF*β* in periodontal cells [16–19]. Therefore, it is thought that beneficial effects of EMD on periodontal regeneration are mediated, at least in part, by these factors. We therefore studied whether NAMPT regulates the expression of their receptors as a possible mechanism, whereby NAMPT may modulate the actions of EMD. BMP binds to BMPR1, upon which BMPR2 is recruited into the complex, or binds to a preformed complex of BMPR1 and BMPR2, which triggers the activation of SMAD and non-SMAD pathways [21]. Following binding of TGF*β* to TGF*β*R2, TGF*β*R1 is recruited into a heterotetrameric receptor complex and then phosphorylated on serine residues by TGF*β*R2, which also finally results in the activation of the SMAD signaling pathway [20]. As shown in the present and our previous studies, PDL cells express receptors for BMP and TGF*β* [24–26]. EMD increased the constitutive expression of BMPRs and TGF*β*Rs, and this EMD-stimulated receptor upregulation was inhibited by NAMPT. These findings suggest that NAMPT can regulate actions of EMD also at receptor level, as it has recently been shown for interleukin-1*β* and biomechanical forces [24–26].

Furthermore, we sought to identify intracellular mechanisms, whereby the stimulatory effects of EMD are modulated by NAMPT. Our experiments revealed that the inhibition of the EMD-induced VEGF and TGF*β*1 upregulation by NAMPT was JNK-dependent. Moreover, NAMPT inhibited the EMD-stimulated phosphorylation of SMADs and, additionally, inhibited their nuclear translocation. Further experiments are needed to clarify how JNK interferes with SMAD signaling and whether additional signaling pathways are involved.

 The present study also confirms our previous studies on the harmful effects of inflammatory signals on regenerative periodontal healing [24–26]. Moreover, in the present study, we provide original evidence that also systemic factors, such as NAMPT, whose levels are increased in obesity, metabolic syndrome, and diabetes mellitus, may interfere with periodontal healing. Although PDL cells are of utmost importance in periodontal regenerative healing, in a clinical setting, additional cells, such as inflammatory cells, keratinocytes, gingival fibroblasts, and osteo- and cementoblasts, are involved. Further studies should examine whether NAMPT also affects these cells under normal and regenerative conditions. Moreover, in addition to NAMPT, the levels of other adipokines, such as leptin, resistin, and adiponectin, are altered in obesity and periodontal inflammation [53–56]. Future studies should therefore focus on the regulation of periodontal cells and their regenerative capacity by these adipokines.

 In summary, the present study shows for the first time that beneficial effects of EMD on a number of PDL cell functions critical for periodontal regeneration are counteracted by NAMPT. Enhanced levels of NAMPT, as found in obesity and periodontal inflammation, may compromise the regenerative capacity of PDL cells and, thereby, regenerative periodontal healing in the presence of EMD. 

## Figures and Tables

**Table 1:** Regulation of VEGF and TGF*β*1 protein syntheses by EMD and/or NAMPT (100 ng/mL) after 3 d, as analyzed by ELISA. Untreated cells served as control. Mean ± SEM (*n* = 12); *significantly (*P* < 0.05) different from all other groups.

Group	VEGF (ng/10^6^ cells)	TGF*β*1 (ng/10^6^ cells)
Control	1.68 ± 0.08	2.08 ± 0.01
EMD	3.88 ± 0.32*	3.39 ± 0.11*
NAMPT	1.73 ± 0.01	1.68 ± 0.02
EMD + NAMPT	3.07 ± 0.34	2.78 ± 0.06
